# MED4/345: Computer-Administered Formative Quizzes in a Basic Science Course

**DOI:** 10.2196/jmir.1.suppl1.e47

**Published:** 1999-09-19

**Authors:** R Ogilvie

**Affiliations:** ^1^Medical University of South CarolinaCharlestonUSA

**Keywords:** Computer-based Examining, Online Examination, Formative Assessment

## Abstract

**Introduction:**

Computer-administered quizzes were introduced into a Cell Biology and Histology course to provide students a means to assess their progress in the course and faculty the opportunity to monitor students' mastery of the course content.

**Methods:**

The computer quizzes, including graphics, were presented on-line using LXR software (www.lxrtest.com) for specific time periods (7 - 14 days) during the course. The aim of this effort was to provide students formative assessment and assistance with pacing their study of course materials. Each computer quiz consisted of 20 - 30 questions with images. Extra credit was earned for each quiz if 70% of the items were answered correctly. The quizzes were served over the campus network to as many as 70 computer workstations, distributed to various locations in our department and the library. Each quiz was accessible once, by a unique user name and password for each student and a time limit set, allowing up to 90 seconds for each question. Feedback was given to the student for each question; the correct answer and a formative instructional statement intended to reinforce the fact or concept being evaluated. Global feedback on each quiz was provided for the entire class. This feedback was delivered on-line, on the course's web site.

**Results:**

We have found that computer-administered course examinations are an efficient and acceptable means of assessing students' learning formatively. They permit a quick examination of the students' mastery of the course content. Such an approach allows for appropriate feedback to be provided in a timely manner and, if needed, instruction could be modified. No serious problems were encountered during the three years we have administered over 4,000 individual quizzes on-line. Greater than 90% of the students elected to participate in this optional activity with more than 85% receiving extra credit for their overall course grade. The computer quizzes were accepted by the students as a useful activity to pace their study and helpful to provide feedback about their mastery of the course content.

**Discussion:**

We have gained confidence through the experience of administering quizzes on-line over our Local Area Network. Our plans are to develop the quizzes and full examinations to be delivered over the Web. Web-based examining has the obvious advantage of using unlimited and cross-platform workstations. However, we are cautiously optimistic about this approach. We recognise that one of the main obstacles in examining over the Web is the potential of collusion or plagiarism. Students that choose the option to take their examination off-campus cannot be checked if they used course notes and/or other resources to answer the questions during the exam. Thus, the control of examination behavior is potentially a serious problem to overcome.

